# Clinical Trial: Heme Arginate in patients planned for Cardiac Surgery (HACS)

**DOI:** 10.1186/1749-8090-10-S1-A69

**Published:** 2015-12-16

**Authors:** Fiona Duthie, Vipin Zamvar, Rachel Thomas, David Kluth, Jeremy Hughes

**Affiliations:** 1 University of Edinburgh/MRC Centre for Inflammation Research, Edinburgh, EH16 4TJ, UK; 2 Department of Cardiothoracic Surgery, Royal Infirmary of Edinburgh, EH16 4SA, UK

## Background/Introduction

Acute kidney injury (AKI) is a significant complication of cardiac surgery and is associated with increased morbidity and mortality [1]. Despite much research, there is no specific therapy available. Although AKI can be multifactorial, ischaemia reperfusion injury (IRI) often plays a key role. Thus, cardiac surgery offers an attractive opportunity for translational AKI research given the predictive haemodynamic challenge to renal perfusion.

Hemeoxygenase-1 (HO-1) is a key inducible anti-inflammatory enzyme that catalyses the breakdown of the pro-oxidant protein heme ubiquitously found at inflamed sites. The drug heme arginate has been in use for over 20 years in the treatment of porphyria but also upregulates HO-1 in peripheral blood mononuclear cells (PBMCs) [2] and ameliorates calf muscle ischaemia [3]. In addition, treatment of mice with heme arginate prior to renal IRI strongly upregulates renal HO-1 expression and protects from AKI [4]. We therefore hypothesise that HA may offer a prophylactic therapy for human renal IRI via the upregulation of HO-1.

## Aims/Objectives

The HACS Trial aims to determine whether heme arginate will upregulate HO-1 in PBMCs in patients aged 60 or above who are scheduled for cardiac surgery, and to verify its safety in this patient cohort.

## Method

20 participants, who are scheduled for elective cardiac surgery, will be randomised to receive 1 mg/kg or 3 mg/kg heme arginate. The primary end point will be the difference in PBMC HO-1 protein from baseline at 24 hours. Secondary end points include HO-1 gene expression, safety and HO-1 genotype.

## Results

Results are expected in July 2015. At the time of abstract submission, 14 of 20 participants have been recruited.

## Discussion/Conclusion

Data from the HACS trial will inform a subsequent multicentre randomised controlled trial of heme arginate versus placebo in the prevention of AKI in patients deemed to be at higher risk of developing AKI post cardiac surgery

## References

[B1] Thakar CV Worley S Arrigain S Yared JP Paganini EP Influence of renal dysfunction on mortality after cardiac surgery: modifying effect of preoperative renal function Kidney Int 2005 67 3 1112 1119 15698452 10.1111/j.1523-1755.2005.00177.x

[B2] Doberer D Haschemi A Andreas M Zapf TC Clive B Jeitler M Haem arginate infusion stimulates haem oxygenase-1 expression in healthy subjects. Br J Pharmacol 2010 161 8 1751 1762 20718734 10.1111/j.1476-5381.2010.00990.x PMC3010580

[B3] Andreas M Schmid AI Doberer D Schewzow K Weisshaar S Heinze G Heme arginate improves reperfusion patterns after ischemia: a randomized, placebo-controlled trial in healthy male subjects J Cardiovasc Magn Reson 2012 14 55 22857721 10.1186/1532-429X-14-55 PMC3438022

[B4] Ferenbach DA Nkejabega NC McKay J Choudhary AK Vernon MA Beesley MF The induction of macrophage hemeoxygenase-1 is protective during acute kidney injury in aging mice Kidney Int 2011 79 9 966 976 21248714 10.1038/ki.2010.535

